# Risk and Response-Adapted Treatment in Multiple Myeloma

**DOI:** 10.3390/cancers12123497

**Published:** 2020-11-24

**Authors:** Titouan Cazaubiel, Olga Mulas, Lydia Montes, Anaïs Schavgoulidze, Hervé Avet-Loiseau, Jill Corre, Aurore Perrot

**Affiliations:** 1 Hematology Department, University Hospital, 33600 Bordeaux, France; titouan.cazaubiel@chu-bordeaux.fr; 2 Centre de Recherche en Cancérologie de Toulouse, Institut National de la Santé et de la Recherche Médicale U1037, 31059 Toulouse, France; anais.schavgoulidze@live.fr (A.S.); AvetLoiseau.Herve@iuct-oncopole.fr (H.A.-L.); perrot.aurore@iuct-oncopole.fr (A.P.); 3 Hematology Unit, Businco Hospital, Department of Medical Sciences and Public Health, University of Cagliari, 09124 Cagliari, Italy; mulasolga@unica.it; 4 Hematology Department, University Hospital, 80054 Amiens, France; Montes.Lydia@chu-amiens.fr; 5 Unit for Genomics in Myeloma, Institut Universitaire du Cancer de Toulouse-Oncopole, University Hospital, 31059 Toulouse, France; 6 Hematology Department, Institut Universitaire du Cancer de Toulouse-Oncopole, University Hospital, 31059 Toulouse, France

**Keywords:** multiple myeloma, cytogenetics, response-adapted treatment, personalized therapy

## Abstract

**Simple Summary:**

Therapeutic strategies in multiple myeloma have been adapted only to age and comorbidities for a long time. Given the currently available therapeutic and technologic arsenal, the time may have come to refine this adaptation. First, high-risk patients should benefit from the most intensive and efficient combinations from diagnosis. Here, we review these different strategies and how to define and identify high-risk myeloma patients in current clinical practice. In addition, the advent of technologies detecting minimal residual disease gives us this opportunity to define the quality of response to treatment with an unpreceded sensitivity and adapt treatment accordingly. Finally, even if molecular targeting is still nascent in myeloma, some molecular features are interesting to detect at relapse to determine optimal salvage treatments.

**Abstract:**

Myeloma therapeutic strategies have been adapted to patients’ age and comorbidities for a long time. However, although cytogenetics and clinical presentations (plasmablastic cytology; extramedullary disease) are major prognostic factors, until recently, all patients received the same treatment whatever their initial risk. No strong evidence allows us to use a personalized treatment according to one cytogenetic abnormality in newly diagnosed myeloma. Retrospective studies showed a benefit of a double autologous transplant in high-risk cytogenetics according to the International Myeloma Working Group definition (t(4;14), t(14;16) or del(17p)). Moreover, this definition has to be updated since other independent abnormalities, namely gain 1q, del(1p32), and trisomies 5 or 21, as well as TP53 mutations, are also prognostic. Another very strong predictive tool is the response to treatment assessed by the evaluation of minimal residual disease (MRD). We are convinced that the time has come to use it to adapt the strategy to a dynamic risk. Many trials are ongoing to answer many questions: when and how should we adapt the therapy, its intensity and duration. Nevertheless, we also have to take into account the clinical outcome for one patient, especially adverse events affecting his or her quality of life and his or her preferences for continuous/fixed duration treatment.

## 1. Introduction

Multiple myeloma (MM) is a plasma cell dyscrasia that is involved in approximately 15% of all hematological malignancies. Most patients are over 65 years old, with a median age at diagnosis of 70 years old [1]. The availability of new therapeutic classes, such as proteasome inhibitors, immunomodulatory drugs, and monoclonal antibodies, now used in combination, have led to great progress in the management of MM patients, resulting in the improvement of progression-free survival (PFS) and overall survival (OS). However, MM still remains an incurable disease and, actually, the identification of high-risk patients is the main target. Nowadays, the therapeutic decision-making process in MM is based on a fine balance between patients’ characteristics and disease-related biological features. There is generally no clear consensus on the use of tools and, even if a personalized and tailored approach were advisable, the definitive choice is not much yet influenced by recent discoveries.

## 2. Lessons from the Past: Consider the Risk at Diagnosis

### 2.1. Age and Vulnerability

For a long time, patients’ age has influenced the treatment decision-making process, as advanced age increases the incidence of comorbidities and frailty risk. However, age and frailty are not equivalent. There is no universally accepted definition of frailty. According to the phenotypic approach, it is commonly assessed by the presence of a reduction in three out of the following five elements: weight, walking speed, endurance, self-reported energy, and physical activity [2]. Another approach allows the use of a broad number of objective or subjective measures, as long as they are linked to aging. In this way, frailty is a deficit accumulation that increases with age, in which the cumulative presence of aging-associated deficits yields a frailty index [3]. Improving outcomes requires the development of validated MM-specific tools that globally assess frailty in this heterogeneous older population and stratify patients by risk to guide the therapeutic approach and set the treatment goals. Currently, the International Myeloma Working Group Frailty Score (IMWG FS) represents a standard approach to define frail and risk populations in MM. It categorizes patients into fit, intermediate, and frail using age, medical comorbidities, and disabilities [4]. Another type of score is the revised Myeloma Comorbidity Index (R-MCI) based on the Karnofsky Performance Status (KPS), the presence of impaired lung and renal function, frailty, age, and cytogenetic abnormalities [5]. However, when they were compared in a prospective study, little agreement was found between these models [6]. It is difficult to fully appreciate the size of treatment efficacy, as elderly and frail patients are less likely to be included in clinical trials and may receive fewer novel agents, partly as a consequence of comorbidities, polypharmacy, and more rapid physiological decompensation associated with both disease- and treatment-related complications [7]. Real-world data will help to better describe this very particular population of frail patients.

### 2.2. Aggressive Clinical Presentations

Among the different plasma cell dyscrasias, extramedullary presentation (EM) and plasma cell leukemia (PCL) are considered high-risk diseases [8,9]. PCL is characterized by a higher prevalence of adverse clinical and laboratory features as compared to MM, and by an elevated genomic instability, as shown by an increased number of cytogenetic aberrations and other molecular lesions at diagnosis [10]. Therefore, additional worse forms have been identified and, recently, circulating plasma cell levels over 5% have been associated with more aggressive conditions [10,11,12]. A lack of protocol inclusion is a reason for the lack of information about the best treatment choice and, just recently, two prospective trials have demonstrated the feasibility of the inclusion of bortezomib (V)-based and lenalidomide (R)-based regimens [13,14]. In young patients, the combination of bortezomib or thalidomide (T) with high-dose chemotherapy as hyper-CVAD-VD or VTD/VRD/KRD-PACE, may be envisaged [15]. Given that autologous stem cell transplantation (ASCT) is the most appropriate therapeutic choice for eligible patients, the role of tandem ASCT or alloSCT is still debated [16]. Other prognostic features related to the malignant clone are the proliferation index (PI) [17] and the plasmablastic morphology [18], both associated with frequent adverse cytogenetic lesions.

### 2.3. The Huge Prognostic Value of Cytogenetics

High-risk myeloma is classically defined as the presence of at least one of the following: del17p, or translocations of chromosomes 4, 16, or 20 involving the immunoglobulin heavy chain locus: t(4;14), or t(14;16) or t(14;20) determined by Fluorescent In Situ Hybridization (FISH) [19]. Among these high-risk chromosomal changes, the most impactful is undoubtedly the 17p deletion (del(17p)) [20,21]. However, the threshold prognostic value of the clonal size can range from 1% to 60% from one study to another [22,23]. A recent meta-analysis of European data showed a significant clinical impact of the subclonality value of 55–60% by FISH [24]. Genetic defects in 17p are complex and include either a deletion—more or less the length of the short arm of chromosome 17—alone or only a mutation in one allele of the TP53 gene or a biallelic inactivation of TP53, the so-called double-hit myeloma entity, described as displaying an extremely poor prognosis [25]. Nevertheless, even if less of a risk than TP53 bi-allelic inactivation, a del(17p) without a TP53 mutation must be considered as a high-risk abnormality [26]. Some translocations involving the immunoglobulin heavy chain gene locus are also associated with poor outcome in MM. The t(4;14) is more frequent and considered as an MM-specific alteration with a prognostic impact influenced by the presence of other alterations [27,28]. Although the independent prognostic value of t(14;16) has not been clearly demonstrated, it is incorporated in the revised International Staging System prognostic model (R-ISS), along with del(17p) and t(4;14) [29]. Other chromosomal alterations include del(1p32) and a gain of 1q21. The former was observed in 7–8% of the patients at diagnosis and its prognostic impact may be almost similar to del(17p) [30]. The gain of the long arm of chromosome 1 is the most frequent adverse cytogenetic lesion, displayed by one third of newly diagnosed MM cases. Some recent data suggest that only an amplification (more than three copies) would be of high risk, but this will have to be confirmed in prospective studies [31].

Currently, the IMWG recommends the detection of t(4;14), t(14;16), and del(17p) [32]. However, co-occurrence and clonality may significantly affect the weight of each of these factors. A study of the IFM (Intergroupe Francophone du Myélome) group performed a multivariate analysis of a large cohort of patients by SNP (Single Nucleotide Polymorphism) array and FISH. Five abnormalities associated with a shorter overall survival were identified: del(17p), del(1p32), gain 1q, t(4;14), and trisomy 21, and trisomy 5 was protective. A prognostic score including these six factors was developed, each one being associated with a specific weighted prognostic value. The ability of this score to identify prognostic subgroups has been validated in a cohort of patients treated with current first-line therapeutic approaches and a better discriminatory performance than R-ISS was observed [28]. The Mayo Stratification of Myeloma and Risk-Adapted Therapy classification (mSMART), updated in 2013, is not a prognostic score per se, but a tool to stratify patients in order to offer them the most suitable treatment [33].

In clinical practice in 2020, we aimed to combine clinical presentation aspects, biological characteristics, and cytogenetics, but also TP53 mutations, in order to better define the risk of myeloma at initial assessment (Table 1).

### 2.4. What Impact on the Choice of Treatment?

Since the definitions of “high-risk myeloma” differed among studies, comparing their outcome is very complicated. Initially, bortezomib regimens represented the best choice for high-risk patients [34,35]. The role of the second generation proteasome inhibitor carfilzomib (K) has been investigated after interesting results were obtained in relapsed high-risk patients [36]. More recently, phase 3 trials enrolling relapse/refractory patients have addressed the issue of the outcome of del(17p) subgroup patients, finding a gain in PFS in patients treated with elotuzumab [22] and ixazomib [37]. Based on synergistic effects, proteasome inhibitors and IMiDs (Immunomodulatory Drugs) as combination therapies seem to be an effective approach among patients with high-risk cytogenetics [38]. The recent analysis of a large cohort of high-risk patients treated frontline with KRd showed the achievement of MRD negativity in about 50% of the patients and the reduction in risk of early relapse in a group of transplanted patients [39]. The role of the addition of daratumumab in induction and consolidation therapy before and after in autologous eligible patients has been evaluated in several randomized studies. The addition of daratumumab to VTD in the CASSIOPEIA trial has been shown to increase response rates and prolong PFS in high-risk cytogenetics patients, but the worse prognosis was not abrogated [40,41]. Moreover, even if the role of ASCT in eligible patients has been confirmed, the interest of double ASCT is still debated. The BMTCTN0702 trial failed to demonstrate advantages in PFS in double transplant both for standard and high-risk patients [42]. In contrast, recent data from the EMN02/HO95 study showed significant improvement in 5-year PFS and OS in double compared to single ASCT. Specific analysis conducted in high-risk cytogenetics, including the del(17p) subgroup, found similar evidence [43]. Extended consolidation therapy with RVd (Revlimid Velcade dexamethasone) regimens after autologous transplantation has also been evaluated, with improved achievement in Very Good Partial Response VGPR and OS [44].

In a non-eligible ASCT setting, triplets were superior to doublets in the presence of high-risk cytogenetics. In the VISTA trial, the bortezomib melphalan prednisone (VMP) showed no differences in terms of complete response, time to progression, or OS between standard and high-risk patients, unlike the MP arm. On the other hand, adding thalidomide to MP improved the response rates for ISS stage III patients. Unfortunately, an increase in adverse events was reported in both studies [45,46]. The main goal in these patients should be to find a balance between toxicity and therapeutic benefits; hence, recent data have shown that molecular events have a larger effect on outcome in younger patients, with their relative contribution diminishing in the elderly [47]. This problem could be overcome through modifications in dose and schedule, without compromising efficacy. A phase 2 study of a re-modulated dosage of RVd (RVd-lite) showed comparable PFS and OS with fewer treatment-related symptoms [48]. The introduction of novel agents, such as daratumumab, in classical treatment has shown interesting results. Indeed, the combination with VMP followed by daratumumab maintenance, in the ALCYONE trial, significantly improved PFS compared to VMP alone (HR 0.50; 95% CI 0.38–0.65; *p* < 0.001) after a median follow-up of 16.5 months. The advantage was evident in patients older than 75 years, ISS stage III, or high-risk cytogenetics [49]. In addition, the risk of disease progression or death was significantly lower among patients receiving daratumumab plus lenalidomide and dexamethasone than among those who received lenalidomide and dexamethasone alone [50].

## 3. The Near Future (Tomorrow): Adapt the Strategy to the Response and the MRD

In the last two decades, therapeutic advances have improved the prognosis of MM patients [51,52]. A better risk stratification allowed a better use of this currently available therapeutic arsenal and contributed to this improvement. Nevertheless, MM patients’ outcomes remain heterogeneous with different response depth and duration. Response depth, such as achieving complete remission (CR), is a significant prognostic factor and is associated with better outcomes [53]. Therefore, response-adapted treatment strategies have emerged.

Nowadays, according to international recommendations [54,55,56], patients with less than a very good partial response (VGPR) after induction could benefit from a tandem intensification. Similarly, some clinical trials investigate a response-adapted approach. The Myeloma XI trial [57] showed that sequential treatment with bortezomib, cyclophosphamide, and dexamethasone (VCD) for patients not achieving at least VGPR after IMiD-based triplet induction improved the response rate and PFS. More recently, in the MM5 trial [58], cessation of lenalidomide maintenance therapy for patients achieving a CR was associated with a shortened OS compared to a fixed-duration maintenance of 2 years. However, these treatment strategies based on conventional response criteria have severe limitations. First, with the new effective drug combinations, more than 50% of MM patients achieve a CR [39,40,41,50]. Thus, the CR endpoint for response-adapted treatment seems to be less useful. Then, patients with high-risk cytogenetics abnormalities achieve similar CR rates than other patients but have paradoxically inferior survival. Finally, despite achieving a CR and even stringent complete response (sCR), most patients relapse, reflecting a persistent disease undetectable with standard methods, namely the minimal residual disease (MRD). To define further and deeper responses beyond CR, the International Myeloma Working Group defined new criteria for response and MRD assessment in 2016 [59]. According to these criteria, MRD should be assessed in the bone marrow either by next-generation flow cytometry (NGF), or by next-generation sequencing (NGS), by measuring, respectively, the patient-specific aberrant phenotypes or clonal rearrangements of the immunoglobulin genes. Both methods have advantages and disadvantages; most importantly, both are theoretically capable of achieving a sensitivity of 10^−6^, although this is probably more difficult in the routine for flow cytometry.

Two meta-analyses [60,61] supported the use of MRD for response monitoring in MM by showing a beneficial impact of undetectable MRD on patient outcomes, with better OS and PFS. Moreover, undetectable MRD surpassed other traditional response criteria. Patients with CR and positive MRD had similar outcomes compared to patients with partial response or VGPR and positive MRD, highlighting the fact that the prognostic value of CR was related to undetectable MRD [62,63]. This prognostic impact of undetectable MRD supported the incorporation of MRD assessment in clinical trials as an endpoint to compare therapeutic strategies. For example, in the first line, the IFM2009 trial [64,65] compared a triplet regimen (bortezomib, lenalidomide, dexamethasone) with or without intensification. Undetectable MRD was a strong prognostic factor for PFS and OS in both arms. The proportion of patients achieving undetectable MRD was higher with intensification, but PFS was similar in patients achieving undetectable MRD irrespective of the treatment received. 

In addition to its prognostic impact, MRD highlighted the notion of dynamic risk in MM. Indeed, achieving undetectable MRD may also abrogate some adverse risk factors, such as high-risk cytogenetics and ISS, as shown by Perrot et al. [65], or R-ISS, as shown by [66]. Patients displaying these adverse factors and who achieve undetectable MRD, although it is less likely (in particular in the del(17p) subgroup), have similar outcomes than standard-risk patients with undetectable MRD.

Consequently, since MRD is one of the most relevant prognostic factors and allows for the assessment of dynamic risk, new response-adapted treatment strategies based on MRD have emerged. Different clinical trials are ongoing to assess if MRD could help in clinical decision-making at various stages of treatment. 

### 3.1. Induction/Intensification

Nowadays, after induction, intensification with ASCT is performed in the first line for eligible patients regardless of the depth of pre-transplant response. In the IFM2009 trial [65], patients achieving undetectable MRD showed similar survival irrespective of the use of intensification. Therefore, MRD evaluation after induction could help to determine which patients will benefit more from intensification. For patients achieving undetectable MRD, one can wonder about the interest of delaying its use for relapse. The role of tandem intensification can also be challenged for patients not achieving undetectable MRD. The future IFM 2020 trial will answer these questions by randomizing eligible patients with undetectable MRD between immediate intensification or only extended consolidation. Patients with detectable MRD will receive either simple intensification with standard consolidation or tandem intensification.

### 3.2. Consolidation/Maintenance

In standard clinical practice, patients receive consolidation and maintenance to improve disease responses after ASCT. As for intensification, some patients achieving deep response levels may not need further treatments. 

The phase 2 trial MASTER (NCT03224507) assessed MRD-based response-adapted therapy. Therapy consisted of four cycles of daratumumab-carfilzomib-lenalidomide-dexamethasone (Dara-KRD) as induction, intensification with ASCT, and zero to eight cycles of Dara-KRD consolidation. MRD was evaluated at each treatment phase, and patients reaching two consecutives undetectable MRD analyses received no further therapy and started a treatment-free observation. The initial results showed that this approach is feasible and leads to high response rates. Similarly, we could consider response-adapted strategies using more intensive regimens, such as bispecific antibodies or CAR (Chimeric Antigen Receptor) T-cells, for patients not reaching undetectable post-transplant MRD.

Moreover, controversies remain over the optimal duration of maintenance therapy with a fixed-duration or treat-to-progression approach. Studies are ongoing to determine whether MRD status can guide the length of post-ASCT maintenance. For instance, the DRAMMATIC/SWOG S1803 study (NCT04071457) compare daratumumab and lenalidomide to lenalidomide as post-transplant maintenance and use MRD to direct therapy duration. After two years of undetectable MRD, patients are randomized to either continue or discontinue the treatment. If these approaches are successful, it will be important that have less invasive methods to assess MRD. The field of liquid biopsy, if adapted to MM, could meet this need (see below).

### 3.3. Relapse Treatment

As well as guiding first-line treatment, MRD might inform retreatment at time of relapse. Indeed, since early intervention at relapse seems to benefit patients in terms of survival [67], relapse treatment strategies leveraging on MRD have emerged. In the REMNANT trial (NCT04513639), after conventional first-line therapy, patients achieving undetectable MRD are randomized to either start treatment as soon as MRD becomes detectable or as the disease progresses according to IMWG criteria. For some specific patients, MRD could also guide the duration of relapse treatment, thus facilitating treatment-free intervals.

However, MRD-based response-adapted therapy faces some limitations. First, achieving undetectable MRD is not the same as a cure, in particular when the sensitivity is only 10^−5^. For instance, Raje et al. [68] reported a very high rate (94%) of early undetectable MRD of patients treated with anti-BCMA (B-cell maturation antigen) CAR-T cells. However, most patients relapsed early with a median PFS of 12 months. This highlights the importance of achieving a sustained undetectable MRD, defined by undetectable MRD with a minimum of a one-year interval. In the FORTE trial [39] comparing carfilzomib-lenalidomide-dexamethasone (KRD) induction followed by intensification and ASCT to KRd alone, patients achieved high rates of undetectable MRD at 10^−5^ regardless of treatment. However, patients with KRD-ASCT presented a higher rate of sustained MRD negativity at one year, especially high-risk patients. These results point out the risk of treatment strategy based on a snapshot in time and unveil the question of the timing of MRD assessment. The issue of the optimal threshold for MRD negativity is still debated, even if everyone agrees that the highest sensitivity is the most discriminant, whatever the methods used. Nowadays, the threshold is 10^−5^ to 10^−6^, but lower response detection might reveal different clinical outcomes and so change the treatment strategies.

Secondly, current MRD methods might not always provide a reliable assessment of residual disease. Indeed, the sensitivity of MRD assessment in the bone marrow by NGF or NGS relies on the sample quality. For instance, hemodilution can lead to an underestimate of residual MM cells and false-negative results. Furthermore, MM presents a spatial heterogeneity with patchy bone marrow infiltration and potential extramedullary disease. Recently, the CASSIOPET trial [69] demonstrated the potential bias of this spatial heterogeneity by evaluating residual disease with MRD and PET-CT (Positron Emission Tomography and Computed Tomography). Among patients achieving undetectable MRD, 10% might still present residual disease with PET-CT positivity. Therefore, better response assessment may necessitate the combination of “biological MRD” with imaging, but further studies are needed. Similarly, MRD detection in peripheral blood (liquid biopsy) could be an attractive additional method. Nevertheless, with current technologies, the sensitivity does not equal the one that can be obtained in the bone marrow, and no correlation was obtained between circulating tumor DNA and bone marrow for MRD using only immunoglobulin gene rearrangements [70], suggesting that it may not guide the treatment alone.

Finally, some (rare) patients with positive MRD may also have prolonged long-term survival. In an elderly population, Rodríguez-Otero et al. [71] showed that the MGUS (Monoclonal Gammopathy of Undetermined Significance)-like phenotypic signature was a potential post-treatment biomarker of long-term disease control. Interestingly, the benefit of the MGUS-like profile in long-term survival is independent of MRD. These results suggest that pursuing undetectable MRD might not be necessary for all patients. Like the MGUS-like signature, the immune profile might help to distinguish which patients will benefit from undetectable MRD.

## 4. In the Long Run (after Tomorrow): A Combined Approach Including Molecular Characteristics?

What about targeted therapy in MM? This approach is very widespread in solid tumors. Though there are some undeniable successes of targeted therapy in other hematological malignancies, such as acute leukemias, some specific features make the task difficult in MM. Data obtained from NGS have shown that no single unifying mutation has been found in MM, and that mutational landscape heterogeneity is very high [31,72,73,74]. The median number of mutations per transcribed MM genome is approximately 60 [72]; compared to other cancers, MM is in the middle, with more mutations than acute leukemia but fewer than carcinogen-induced tumors [75]. The most frequently mutated genes are *KRAS* and *NRAS* (approximately 25% and 20% of patients, respectively) followed by *DIS3*, *FAM46C*, *BRAF, TP53*, and *TRAF3*. All other mutations are observed in less than 5% of patients [73,76,77]. In practice, very few studies have reported targeted treatment in MM. The activating BRAF V600E mutation has been specifically targeted in a single patient with vemurafenib [78], with encouraging data, although very advanced disease. However, this targetable V600E mutation represents only a subset of *BRAF* mutations in MM. An unresolved issue in the field of targeted therapy is the minimum allelic fraction required for a mutation to be targeted. Indeed, data from exome analysis reveal that in many cases, MM driver mutations are present only in subclones [73,74,76,77,79], preventing the use of a specific inhibitor in monotherapy. The combination of inhibitors targeting mutations occurring in different subclones could be effective if cumulative toxicities are acceptable. The use of MEK (Mitogen-activated Extracellular signal-regulated Kinase) inhibitors has been proposed in patients displaying *KRAS* or *NRAS* mutations, but the results were unfortunately not very convincing [80,81,82,83]. The only mutation with well-established prognostic value implies the tumor suppressor p53 [77,79] but, here, again, its therapeutic restoration is challenging [84]. The prospect of a targeted treatment of MM based on mutations therefore remains uncertain.

The only current convincing targeted therapy in MM, the BCL2 inhibitor venetoclax, actually does not have a clear target [85]. This drug has been associated with 40% of objective responses in multi-refractory patients harboring the t(11;14) translocation in a single agent trial [86]. However, no biological link between *CCND1* and *BCL-2* has been clearly highlighted. The presence of t(11;14) may only be an imperfect surrogate marker of high BCL2 expression, with some patients without t(11;14) also displaying good-quality response, and some with t(11;14) being refractory. The combination of venetoclax with bortezomib and dexamethasone shows promising efficacy in relapsed refractory MM [87], even if preliminary results of the double-blind randomized placebo-controlled BELLINI trial led to a warning from the FDA due to an excess of infectious deaths in the venetoclax arm [88]. Nevertheless, venetoclax-based strategies should be considered at relapse in patients harboring t(11;14), taking into account the benefit/risk balance and under the use of infectious prophylaxis.

Beyond targeted therapy in the strictest sense, some drugs may be particularly efficient in specific cytogenetic profiles. The most striking example is t(4;14). Various clinical data have suggested that bortezomib is of particular interest in patients with t(4;14) [46,89,90]. The widespread use of this drug has probably contributed to reducing its unfavorable prognosis [91]. Carfilzomib and ixazomib have also demonstrated their ability to increase PFS in combination with lenalidomide and dexamethasone in high-risk patients [92]. Nevertheless, large studies devoted specifically to patients harboring t(4;14) are lacking. Current real-world studies could probably evaluate if proteasome inhibitor-based combination should be systematically used in such patients. For patients harboring del(17p), there is less data, but pomalidomide may be of specific interest [93]. A phase 2 trial specifically dedicated to patients harboring either t(4;14) or del(17p) is currently evaluating a combination of ixazomib, pomalidomide, and dexamethasone (IFM 2014-01, NCT03683277).

Apart from cytogenetic and molecular abnormalities, some clinical presentations observed at relapse, such as secondary plasma cell leukemia and extramedullary localizations, may benefit from certain specifically oriented strategies. Some authors have suggested adapting treatment in patients displaying a plasmablastic morphology and a high PI using anti-proliferative drugs, such as anthracyclines and inhibitors of aurora kinase [17,94].

Given their very poor prognosis, cases with TP53 biallelic inactivation (double-hit myeloma) may represent a totally unmet medical need [25]. Novel agents as idasanutlin or flotetuzumab have been investigated in acute myeloid leukemias with TP53 abnormalities [95,96]. Such approaches targeting MDM2 (a negative regulator of tumor suppressor p53) and/or characterizing the impact of the immune landscape on the drug’s resistance might be also helpful in MM. In the future, risk factors related to the myeloma microenvironment, such as immunoparesis, may also be taken into account [97].

## 5. On the Finish Line, the Clinical Pragmatic Approach

Emerging therapies and improvement in risk and response assessment have increased myeloma patients’ survival, but also the complexity of treatment choice. To ease this decision, new treatment strategies based on a better risk stratification, a better response assessment, and the discovery of molecular characteristics have and will emerge. These strategies rely on clinical trial results under ideal circumstances. However, their application in routine clinical practice might face some real-life limitations and thus needs to integrate a more pragmatic approach.

First, since more patients presented prolonged survival, quality of life (QoL) should be an essential consideration in treatment strategy. With advances in the management of MM, clinical outcome benefits resulted in an improved QoL for myeloma patients [98]. However, multidrug combination and continuous or long-term treatment for maintenance and relapses become preponderant and expose patients to more potential side effects. Thus, toxicity will impact treatment possibility in a significant way, whatever the risk and the response. Indeed, even with new therapeutic agents, between 5% and 30% of patients discontinued maintenance treatment due to toxicity [57,99,100]. Further, few clinical trials included extended follow-up of QoL and assessed only disease and treatment symptoms. Other dimensions like psychological impact, social and family situation, treatment access, or economic consideration may impact the treatment decision. In an exploratory study, Hulin et al. [101] highlighted the impact of relapse with an emotional, physical, and social burden. Therefore, some patients with fear of relapse may prefer continuous treatment. On the contrary, other patients wishing longer treatment-free intervals may choose fixed-duration treatment. Thus, as equally important as risk and response, the treatment decision should also reflect all these patient-related factors.

**Figure 1:**
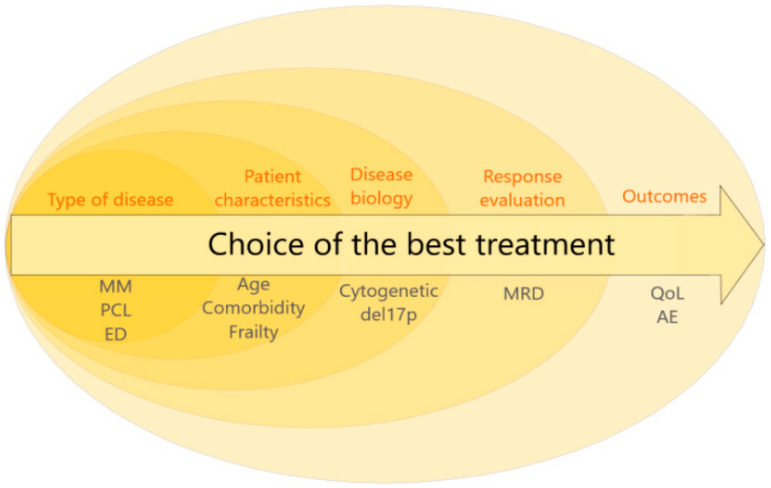
Multiparametric and dynamic model to consider the best treatment choice for one patient. MM: multiple myeloma, PCL: plasma cell leukemia, ED: extramedullary disease, MRD: minimal residual disease, QoL: quality of life, AE: adverse events.

Finally, individualized patient-adapted strategies and those based on risk and response are not opposites but rather complementary. Indeed, better identification of high-risk patients and better detection of the quality of response will facilitate shared decision-making between patient and physician. This new knowledge will help physicians and patients to assess more finely the benefit–risk balance among multiple options and thus to reinforce treatment decisions (Figure 1). In the ever-changing field of myeloma treatment, risk and response assessment is like a compass: it sets the course for treatment decisions, but real-life clinical practice steers the ship.

## Figures and Tables

**Table 1:** Main characteristics used to define high-risk multiple myeloma in 2020.

**Clinical–biological presentation**	Plasma cell leukemia
	Extramedullary presentation
	Plasmablastic cytomorphology
**Cytogenetics**	del17p
	t(4;14)
	t(14;16)
	t(14;20)
	del(1p32)
	Gain of 1q21 (three or more copies)
**Mutations**	TP53
**Prognostic scores**	R-ISS
	IFM multiparametric score
	mSMART

(R-ISS = Revised International Staging System, IFM = Intergroupe Francophone du Myélome, mSMART = Mayo Stratification of Myeloma and Risk-Adapted Therapy).
